# Establishing a non-viral transfection system for fibroblasts using branched polyethylenimine

**DOI:** 10.1371/journal.pone.0329666

**Published:** 2025-08-05

**Authors:** Tim Lukas Lübbersmeyer, Kirsten Wissel, Andrea Hoffmann, Lisa Kötter, Thomas Lenarz, Gerrit Paasche

**Affiliations:** 1 Department of Otorhinolaryngology, Head and Neck Surgery, Hannover Medical School, Hannover, Germany; 2 Lower Saxony Center for Biomedical Engineering, Implant Research and Development (NIFE), Hannover, Germany; 3 Cluster of Excellence Hearing4all, Hannover Medical School, Hannover, Germany; 4 Department of Orthopaedic Surgery, Hannover Medical School, Hannover, Germany; Faculty of Medicine of Tunis, TUNISIA

## Abstract

Enhanced survival of spiral ganglion neurons (SGN) could improve hearing in patients with cochlear implants. Supplying these cells with growth factors like brain-derived neurotropic factor (BDNF) has been shown to improve cell survival and vitality. Direct applications of BDNF, e.g., via integrated drug-delivering cannula, elevate the surgical risks, as well as the probability of infections. Therefore, in vivo production of BDNF by on-site transfection of cells with plasmid DNA coding for BDNF might be an option. Polyethylenimine (PEI) was chosen as a non-viral transfection reagent, due to its comparatively low cytotoxicity, ease of preparation and use. NIH/3T3 fibroblasts were used as model cells for fibroblast transfection that could be transferred to cochlear implants. Branched 25 kDa PEI was diluted in PBS and mixed in different ratios with two different plasmids coding for BDNF and tdTomato simultaneously. Particle size and zeta potential were determined, and cell metabolic activity was measured using MTT. Transfection efficiency was determined by counting cells with and without transfection-induced fluorescence. Complexes of DNA and PEI were mostly larger than DNA and PEI molecules alone. In addition, complexation of DNA with PEI altered the surface charge of the particles. The cell metabolic activity test confirmed cytocompatibility for almost all tested complexes of DNA and PEI. Plasmid A, which was based on a lentiviral vector backbone, resulted in a very low transfection efficiency of 0.4%, whereas with plasmid B, which was based on pUC19, a transfection efficiency of about 9% was achieved. Size and zeta potential indicate the formation of complexes with both plasmids. Transfection efficacy appears to be dependent on the size of the DNA molecule used. With successful transformation of nearly 10% efficiency and a comparatively low cytotoxicity, the proposed transfection system using plasmid B may be used for further experiments.

## Introduction

With more than one million successfully implanted patients [1], cochlear implants (CI) have become irreplaceable in the treatment of moderate to profound sensorineural hearing loss. However, since a number of patients show no improvements in speech perception [2], further research to improve cochlear implants is needed. One area for improvements might be the interface between device and neurons, specifically the spiral ganglion neurons (SGN). These cells normally transmit signals from the hair cells to the central nervous system [3], in patients with CI, they receive those signals from the device [3]. It has been shown that the number of SGN in patients with sensorineural hearing loss is significantly lower than in healthy patients [4]. A higher survival of SGN could also result in a better performance of cochlear implants [5,6]. One of the promising strategies to improve the SGN survival is the application of growth factors, like brain-derived neurotropic factor (BDNF). Both, in vitro [7] and in vivo [8] experiments have shown not only an improved SGN survival in presence of BDNF, but further growth of auditory neurons. The axonal growth of SGN follows a concentration gradient of BDNF towards the highest concentration [9], thus providing a possible method not only to preserve SGN, but also to redirect their neurites to close the gap between nerve and device.

Improving cochlear implants to apply BDNF to the cochlea could be a way to enhance device performance without the need for additional interventions. Direct application of BDNF into the perilymph via a drug-delivering cannula integrated into the implant [10] has been shown to increase SGN survival, but due to the associated risks (such as infection, increased risk of shearing-induced damage, limitations on flow rate) [11], this method is only viable for a comparably short period of time. To mitigate the risks, BDNF has been integrated into different possible coatings for CI, e.g., silica nanoparticles [12], and shown to be effective in promoting SGN survival and growths. CI coated with alginate hydrogel [13] have been successfully used in vivo. However, this method is limited to a few months at most.

The production of BDNF on-site would be a different approach using existing cellular structures. Direct injection of a viral vector containing DNA coding for BDNF into the perilymph via a CI with an integrated drug-delivering cannula showed an improved survival of SGN and better CI function [14]. However, this method does not target specific cells. In order to provide also directional cues for neurite growth towards the electrode array, a possible target for transfection would be fibroblasts. Fibroblast growth around the CI is a known reaction of the body to the surgical procedure [15], which leads to an increased post-surgery impedance at the electrode contacts [16]. Although this growth has been successfully reduced, e.g., in guinea pigs by integrating dexamethasone into the silicone of the CI [17], a number of fibroblasts remains and these represent potential target cells for *in vivo* transfection. Seeding of in vitro transfected fibroblasts to produce BDNF on CI electrodes prior to implantation resulted in an increased survival of SGN [18], validating fibroblast as a target for this approach. A transfection vector embedded in the coating of the implant surface could be a promising tool for transfection of endogenous fibroblasts and long-term delivery of BDNF.

Transfection can be achieved with viral or non-viral vectors. Viral vectors can be divided into retroviral vectors (e.g., lentivirus) or non-retroviral vectors (adenovirus and adeno-associated virus) [19]. Since the first use of viral vectors in gene therapy over forty years ago, extensive research has led to a better understanding and several commercially available applications [19]. The advantages of viral transfection systems and the pertinent viruses are a high transfection efficiency, long-term integration into the cells and integration into both dividing and non-dividing cells [20]. Both types of viral vectors share certain drawbacks, mainly a high cytotoxicity [21] and immunogenic responses [22]. Furthermore, non-retroviral drugs are amongst the most expensive drugs on the market [23]. Because of a noticeable lower cytotoxicity and lack of immunogenic responses [21], non-viral transfection was chosen in the current study. Non-viral transfection can be divided into physical transfection such as electroporation and transfection by laser [24] or sonophoresis [25], and chemical transfection [24]. However, physical transfection appears unsuitable for use with cochlear implants. Chemical transfection can be further divided into liposomal and non-liposomal transfection [21]. Liposomal transfection tends to have a higher transfection efficiency [24], but is impeded by a higher cytotoxicity [26] as well as a higher risk of inducing apoptosis and inflammatory cascades [27], which could impede SGN survival. Therefore, polyethylenimine (PEI) as a non-liposomal transfection reagent was chosen.

PEI is an established reagent in transfection [28,29] and has been described as the ‘gold standard’ [24]. It shows overall high reliability, is readily available and affordable, as well as easy to handle [24]. Its cytotoxicity, which has been well documented [30], has successfully been lowered by various modifications, e.g., the addition of specific peptides or polymers [31], and is still comparatively low [21,32]. PEI has already been successfully integrated into polymeric coatings for implantation and subsequent *in vivo* release of DNA-PEI-polyplexes [32]. Whilst linear PEI seemed to be superior *in vitro*, branched PEI showed better results *in vivo* [33]. Although transduction with PEI is not yet commercially available, several clinical trials have used PEI successfully [24]. Especially topical applications of PEI, e.g., direct injection into cancer cells, have been successfully proven [24]. In the current study, NIH/3T3 fibroblasts were chosen as target cells. NIH/3T3 fibroblasts are easy to use, culturally stable [34] and have shown stable release of BDNF after viral transfection [35]. Although this is a commonly used standard cell line, only few reports of PEI-based transfection of NIH/3T3 fibroblasts exist [36,37]. In both studies, the focus was put on the method of PEI transfection, which was then evaluated with different cell types. The current study was set out to establish a reliable, PEI-based transfection system, which can potentially be later included in the coatings of cochlear implants, by investigating the influence of plasmid size and DNA to plasmid ratio on transfection efficiency in NIH/3T3 fibroblasts.

## Materials and methods

### Preparation of the polyethylenimine (PEI) working solution

Branched PEI with a mass of 25 kDa (Sigma-Aldrich, Taufkirchen, Germany) was dissolved in phosphate-buffered saline (PBS, Sigma-Aldrich) at a concentration of 1 mg/ml and sterile filtrated prior to use. Aliquots of the PEI solution were stored at room temperature in darkness.

### Recombinant gene expression vectors

To establish non-viral transfection using PEI-DNA complexes, two plasmids differing in size and origin were included in this study. Plasmid A (pRRL.PPT.SF.hBDNF) represented a recombinant lentiviral vector with 8572 base pairs (bp) in size. The human BDNF *(hBDNF[NM_001709.5])* expression was under control of a spleen focus-forming virus promoter. In addition, the expression of the marker protein tdTomato was mediated by the internal ribosome entry site (IRES). The generation of the gene expression vector was described in the study of Scheper et al., 2019 [38].

In contrast, plasmid B (ID: VB190613−1042gdk, vector name: pRP-CAG.hBDNF) constructed by VectorBuilder GmbH (Neu-Isenburg, Germany) comprised 5590 bp and was derived from the cloning vector pUC19. Expression of hBDNF was driven by CAG promotor (CMV early enhancer fused to modified chicken β-actin promoter), and that of the tdTomato red fluorescent protein was initiated by the linker T2A (self-cleaving 2A peptide from thosea asigna virus).

Both expression vectors contained coding sequences for ampicillin resistance as selection marker.

### Enrichment and extraction of the gene expression vectors

Plasmid B was provided by the manufacturer in a glycerol (15%) stock following transformation to the competent E. coli strain Stbl3. The in-house constructed plasmid A had been transformed to the E. coli strain DH5α for glycerol (40%) stock preparation. Small chunks of the unfrozen glycerol stocks were inoculated into 5 ml Luria broth (LB) medium (Karl Roth GmbH, Karlsruhe, Germany) containing 100 µg/ml ampicillin (Carl Roth GmbH) and pre-cultivated for at least 2 h at 37 °C and 130 rpm. Following transfer to 250 ml LB medium including 100 µg/ml ampicillin, both bacterial suspensions were incubated for further 12–16 h at 37 °C and 110 rpm. Prior to the extraction of the gene expression vectors according to the manufacturer’s instructions (NucleoBond Xtra Midi Kit, Macherey-Nagel GmbH, Düren, Germany), optical density (OD) of the bacterial suspensions was photometrically measured at 600 nm. Both plasmids were reconstituted with bidistilled water, followed by aspirating 2 µl of the dissolved DNA onto the nanoplate for measuring the DNA concentration by using the plate reader Synergy H1 (Biotek GmbH, Bad Friedrichshall, Germany). Restriction digestion was performed by using the endonucleases BamHI, NaeI, SalI and SpeI to ensure the presence and correct size of the inserted sequences coding for hBDNF and tdTomato. For transfection, the plasmid solutions were diluted to 100 µg/ml in PBS at pH 7.4.

### Preparation of plasmid (A and B)/PEI complexes

The stock solutions containing varying amounts of PEI as well of plasmid A or B were diluted in Dulbecco’s modified Eagle’s medium (DMEM, Bio&Sell, Nürnberg, Germany) without fetal calf serum (FCS, Thermo Fisher Scientific, Waltham, USA) supplementation to reach PEI or plasmid concentrations of 250 ng to 2 µg and 250 ng to 1 µg, respectively, in a final volume of 40 µl for each transfection assay (Table 1). Additionally, PEI and both gene expression vectors alone were prepared in DMEM without FCS at the desired concentrations. All assays were incubated for 15 min at room temperature prior to the transfection process induction in the NIH/3T3 cell culture assays.

**Table 1:** Tested ratios of DNA and PEI in [ng/ng].

250/250	250/500	250/1000	250/2000
500/250	500/500	500/1000	500/2000
1000/250	1000/500	1000/1000	1000/2000

First number: amount of DNA; second number: amount of PEI.

### Dynamic light scattering and zeta potential

For determination of particle size and surface charge of the transfection components, their dynamic light scattering properties and zeta potential were measured using a Malvern Zetasizer Nano ZS (Malvern Panalytical, Malvern, UK) with the following parameters: λ = 532/632 nm, capillary cells heated to 25 °C for 60 seconds, maximum 120 runs. Measurements were performed in triplicate in N = 8 independent experiments. The transfection solutions were prepared as described above. As instructed by the manufacturer, a folded capillary cell was filled with bidistilled water and 40 µl of the transfection assays were injected to the bottom of the capillary cell by using a syringe connected to a long bent needle.

### Seeding, cultivation, and transfection of NIH/3T3 cells

The murine NIH/3T3 fibroblasts (ATCC number CRL 1658, German Collection of Microorganisms and Cell Cultures GmbH, Braunschweig, Germany) were seeded in 96-well cell culture plates (Thermo Fisher Scientific) at a density of 1*10^4^ cells/well in 100 µl DMEM supplemented with 10% FCS. Following pre-cultivation for 24 h at 37 °C and 5% CO_2_, the medium was removed and the cells were washed twice with PBS, followed by supplementation with 40 µl of the transfection reagent containing the amounts of DNA and PEI from Table 1 and 100 µl DMEM, and incubation at 37 °C and 5% CO_2_ for 3 h. The transfection procedure was stopped by removing the transfection solution, washing the cells twice with PBS, and adding 100 µl fresh DMEM before further cultivation for 21 h. Therefore, all reported results reflect the status at 24 h after starting the transfection. N = 6 experiments were used for data collection, and each assay was performed in triplicate (n = 3). NIH/3T3 cell culture assays without PEI, plasmids, and DNA/PEI complexes were used as a reference for relative quantification of the transfection samples. Additionally, transfection experiments were also performed using 250 ng, 500 ng, or 1000 ng of DNA without PEI, as well as 250 ng, 500 ng, 1000 ng, or 2000 ng of PEI without DNA as controls.

### Quantification of transfected NIH/3T3 cells in relation to the total cell number

Following transfection, the NIH/3T3 cells were evaluated under the fluorescence microscope (Zeiss Observer Z1, Carl Zeiss AG, Oberkochen, Germany) at the absorption and emission wavelengths λ = 561/572 nm. For quantitative determination of the transfection rate, four sections of each well were selected and digitally photographed using an Axiocam MRm (Carl Zeiss AG). Then, cell nuclei were stained with DAPI (Sigma-Aldrich). The medium in the wells was removed and the cells were washed once with 50 µl containing 1 µg/ml DAPI in methanol, and 100 µl of DAPI-methanol was added before the cells were incubated for 15 minutes at 37 °C and 5% CO_2_. The solution was then discarded and the cells were washed twice with 100 µl PBS. The RoboSoftware (Palm-Zeiss, Munich, Germany) enabled capturing of images exactly at the same positions already used for documentation of transfected cells. Finally, the transfected cells as well as the corresponding DAPI stained nuclei were counted and analysed using the integrated “analyze particle” function of ImageJ (version 1.52p 22 June 2019, imagej.com, National Institute of Health, Bethesda, Maryland, USA) with particle size set to 20–150 pixels. Transfection rates were calculated as the ratio of red fluorescent cells to the total number of DAPI stained nuclei (in %). Each assay was performed at least in triplicate in N = 6 independent experiments.

### MTT metabolic activity assay

MTT (3-(4,5-dimethylthiazol-2-yl)-2,5-diphenyltetrazolium bromide, Sigma-Aldrich) assay was used for relative quantification of the metabolic activity of the NIH/3T3 cells exposed to PEI and the plasmids alone as well as to plasmid-PEI-complexes at different concentrations and ratios. An amount of 1 mg/ml MTT was dissolved in DMEM without phenol red (Sigma-Aldrich) and sterile filtered. The transfection assay was performed as described above (3 h of incubation plus 21 h of further cultivation) and 24 h after starting the transfection, the medium was exchanged with 50 µl MTT solution and incubated for 2 h at 37 °C and 5% CO_2_. Following decanting the MTT-solution and adding 100 µl of isopropanol, the plate was panned to achieve a homogenous coloration in each assay. Absorption was measured at a wavelength of 570 nm using a microplate reader (Synergy H1). Cell culture assays without PEI or DNA-PEI complexes represented the reference (untreated control) and wells (6 wells per plate) containing DMEM without phenol red alone were used as background control. Each assay was performed at least in triplicate in N = 6 independent experiments. For data analysis, the background was subtracted and the optical densities (OD) of the transfection assays were related to those of the reference and calculated in percent (%).

### Statistical analysis

All data achieved from determination of the dynamic light scattering and zeta potential as well the in vitro cell culture assays were presented as mean ± standard deviation (SD). Statistical evaluation was performed using GraphPad Prism 8 (GraphPad Software, LaJolla, USA). Tests for Gaussian normal distribution were performed using either the D’Agostino-Pearson test (if N = 8) or the Shapiro-Wilk test (if N = 6). One-Way-ANOVA (for Gaussian distribution) and the Turkey’s multiple comparisons test, or Kruskal-Wallis test (for non-Gaussian distribution) followed by Dunn’s multiple comparisons test were used for statistical assessment. P ≤ 0.05 was set as threshold for significant differences.

## Results

### Determination of particle size

**Fig 1:**
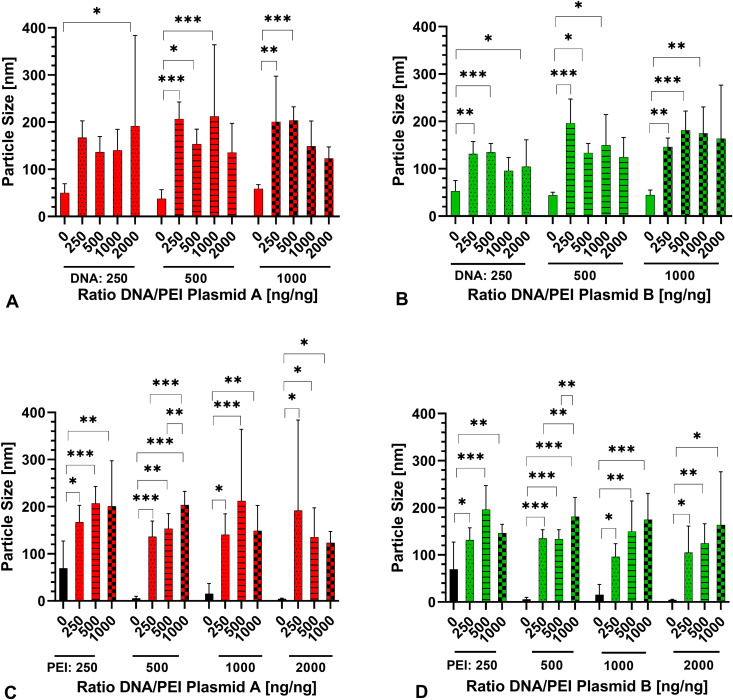
Particle size of complexes of A) plasmid A and B) plasmid B presented as mean ± SD. The amount of PEI [ng] is provided below the columns, the amount of DNA [ng] below the graph. C) plasmid A and D) plasmid B presented as mean ± SD. The amount of DNA [ng] is provided below the columns, the amount of PEI [ng] below the graph. Statistical significance is indicated (*p < 0.05, **p < 0.01, ***p < 0.001). Comparisons were made within the grouped ratios. N = 8 with n = 3. Data in S1 Dataset.

The particle sizes of PBS, PEI, DNA, and their complexes were determined. The mean measured particle size for PBS was 68.15 ± 67.47 nm. Measured sizes for PEI ranged from 4.55 ± 1.29 nm (2000 ng) to 65.37 ± 57.90 nm (250 ng). Particle size for plasmid A was found between 37.61 ± 19.21 nm (500 ng) and 58.93 ± 8.73 nm (1000 ng), whilst those for plasmid B ranged from 44.06 ± 6.47 nm (500 ng) to 52.86 ± 22.22 nm (250 ng). The complexes of plasmid A and PEI ranged between 123.3 ± 24.16 nm and 206.8 ± 36.18 nm. The size of complexes of plasmid B and PEI was between 95.89 ± 28.05 nm and 196.1 ± 50.95 nm (Fig 1).

In complexes of plasmid and PEI, all measured values of complexes were larger than the size of the equal amount of DNA particles (Fig 1). This difference was significant for plasmid A (Fig 1A) assembled with PEI in the complexes for DNA/PEI 250 ng/2000 ng (p = 0.0292), 500 ng/250 ng (p < 0.001), 500 ng/500 ng (p = 0.0167), 500 ng/1000 ng (p = 0.0042), 1000 ng/250 ng (p = 0.0015), and 1000 ng/500 ng (p < 0.001). For plasmid B (Fig 1B), the difference was significant for nearly every ratio tested (ranging from p < 0.001 to p = 0.0265), with only DNA/PEI 250 ng/1000 ng (p = 0.2227), 500 ng/2000 ng (p = 0.1567), and 1000 ng/2000 ng (p = 0.058) not reaching significance.

Likewise, the particle size of the DNA/PEI solution was always larger than the size of an equivalent amount of PEI alone for both, plasmid A (p < 0.001 to p = 0.0261; Fig 1C) and plasmid B (p < 0.001 to p = 0.0394; Fig 1D).

### Determination of zeta potential

**Fig 2:**
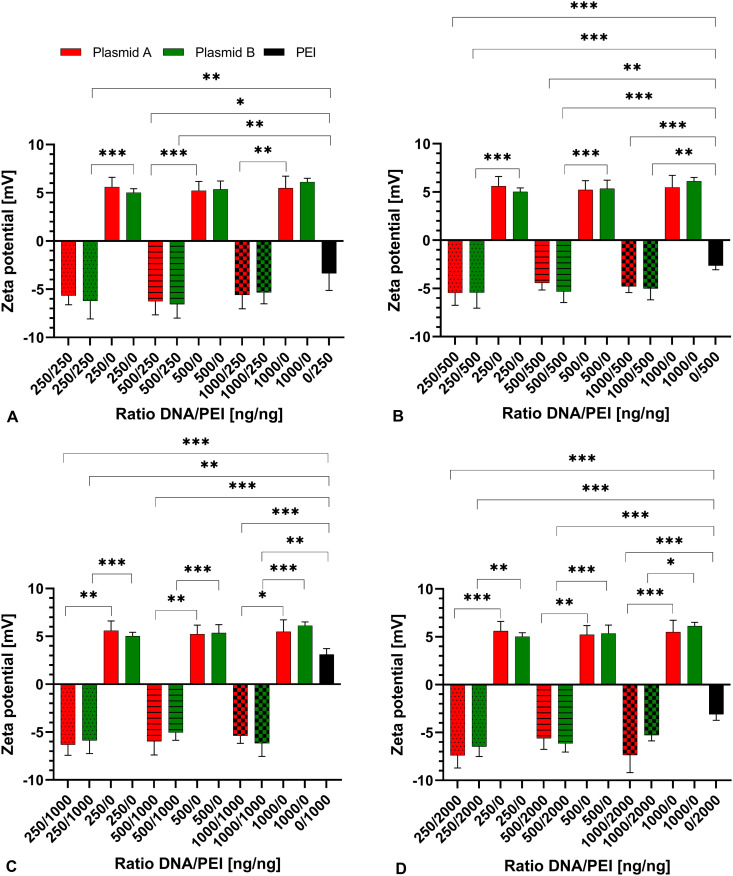
Zeta potential of complexes of plasmid A (red) and B (green) presented as mean ± SD compared to A) 250 ng PEI, B) 500 ng PEI, C) 1000 ng PEI, D) 2000 ng PEI. DNA/PEI ratios [ng/ng] are indicated directly below the columns, plasmid A and B, as well as PEI, are shown in the respective colours. Statistical significance is indicated (*p < 0.05, **p < 0.01, ***p < 0.001). Comparisons were made with the corresponding concentrations of PEI or DNA alone. N = 8 with n = 3. Data in S2 Dataset.

Additionally, the zeta potential was measured in the transfection assays as well as in PEI, DNA, and PBS alone. The zeta potential of PBS was determined to be −1.498 ± 0.192 mV. In PEI, it ranged from −3.362 ± 1.764 mV to 3.103 ± 0.63 mV, with all but one sample (1000 ng PEI) being in the range of −3.363 ± 1.764 mV to −2.638 ± 0.416 mV (Fig 2). This one sample showed an increased number of particles counted (3492 kcps versus 11–250 kcps) compared to the other samples of PEI, whilst also showing more distinct peaks in its results. In plasmid A alone, zeta potential was found between 5.225 ± 0.945 mV and 5.6 ± 1.016 mV with a mean value of 5.437 mV. Plasmid B produced comparable results, ranging from 5.025 ± 0.398 mV to 6.127 ± 0.383 mV with an average of 5.502 mV.

The zeta potential of complexes of plasmid A and PEI was between −4.41 ± 0.746 mV and −7.365 ± 1.826 mV in different concentrations and ratios, with no significant differences between the individual complexes. Using plasmid B and PEI, the zeta potential ranged between −5.01 ± 1.166 mV and −6.55 ± 1.44 mV. Again, there were no significant differences between individual concentrations and ratios.

However, comparing DNA alone and complexes of plasmid A, the charge always changed, with the difference being significant (p < 0.001 to p = 0.0144) except for ratios of DNA/PEI 250 ng/250 ng (p = 0.0705), 250 ng/500 ng (p = 0.1028), 500 ng/500 ng (p = 0.4220), and 1000 ng/500 ng (p = 0.1761) (Fig 2). In plasmid B, this could be observed as well, with the difference reaching statistical significance (p < 0.001 to p = 0.0115) except for DNA/PEI 1000 ng/500 ng (p = 0.1392).

Zeta potential of complexes of plasmid A differed significantly from that of an equal amount of PEI alone in most ratios (p < 0.001 to p = 0.0109), except for ratios of DNA/PEI 250 ng/250 ng (p = 0.1980), 500 ng/2000 ng (p = 0.2410), and 1000 ng/250 ng (p = 0.2738). In plasmid B, this could be observed as well, with statistical significance being reached (p < 0.001 to p = 0.0061) except for ratios of DNA/PEI 500 ng/1000 ng (p = 0.0853), 1000 ng/250 ng (p = 0.0800), and 1000 ng/2000 ng (p = 0.2258).

### Quantitative determination of the metabolic activity (MTT assay)

**Fig 3:**
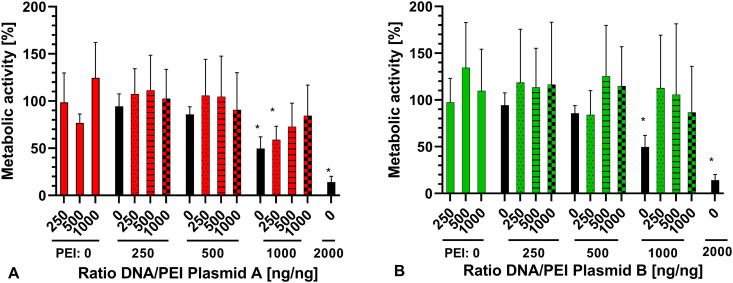
Results of the MTT assay (mean ± SD) of A) plasmid A and B) plasmid B. Amount of DNA [ng] is shown below the columns, amount of PEI [ng] is shown below the graph. Statistical significance against positive control is indicated (*p < 0.05). N = 6 with n = 3. Data in S3 Dataset.

In cells incubated with plasmid DNA alone, the measured rate of cellular activity ranged from 76.73 ± 9.49% to 124.5 ± 37.54% in plasmid A and from 97.40 ± 25.71% to 137.5 ± 48.34% in plasmid B, without an emerging visible trend. In PEI alone, the highest measured rate of cellular activity was 94.25 ± 13.4% with 250 ng PEI (Fig 3). The cellular activity decreased with increasing amounts of PEI, being at its lowest with 14.08 ± 6.18% at 2000 ng PEI. For PEI 1000 ng (p < 0.038) and PEI 2000 ng (p < 0.022), statistical significance compared to the untreated control was found. This resulted in a LD50 of 994 ng PEI per 100 µl.

For the complexes of plasmid A (Fig 3A), the highest measured cellular activity was 111.4 ± 37.2% for the DNA/PEI ratio of 500 ng/250 ng, whilst the lowest was 58.86 ± 14.44% for DNA/PEI 250 ng/1000 ng. Only DNA/PEI 250 ng/1000 ng showed a statistically significant difference to the untreated control (p < 0.047). For plasmid B (Fig 3B), the highest measured cellular activity was 125.2 ± 54.6% for DNA/PEI 500 ng/500 ng, whilst the lowest was 84.15 ± 25.72% for DNA/PEI 250 ng/500 ng. However, there was no statistically significant difference between cellular activity in DNA/PEI complexes and the positive control.

For both plasmids, there were no significant differences between the complexes and the equal amount of DNA alone. However, there was a noticeable trend towards the metabolic activity in complexes being higher than in the equivalent amount of PEI alone. Comparing the respective complexes of both plasmids, there were no significant differences regarding their effects on the metabolic activity of the NIH/3T3 cells.

### Quantitative determination of the transfected NIH/3T3 cells

**Fig 4:**
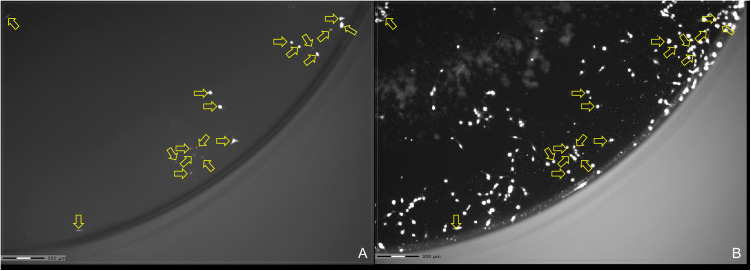
Microscopic images 24 h after start of the 3 h transfection period with Plasmid B (DNA/PEI 250 ng/500 ng). A) Fluorescence signal of tdTomato (excitation/ emission: λ = 561/572 nm). B) DAPI staining of the same section of the culture dish. Fluorescent cells in A) were marked by arrows. The pattern of arrows was then transferred to B).

Control experiments adding PEI or DNA to the cells did not yield any successful transfection evidence by fluorescence of the marker protein, regardless of the amount of substance. In contrast, in all samples treated with DNA/PEI complexes, fluorescent cells were detected (Fig 4).

**Fig 5:**
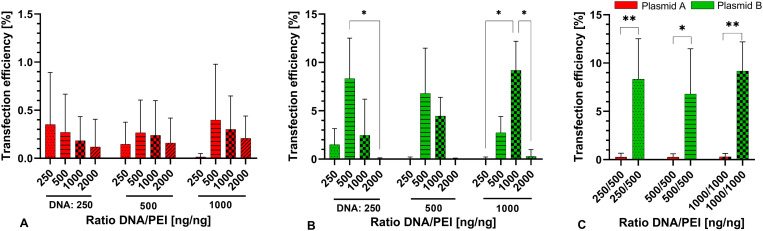
Transfection efficiency (mean ± SD) with the different DNA/PEI ratios in A) plasmid A, B) plasmid B. The amount of PEI [ng] is provided below the columns, the respective amount of DNA [ng] is provided below the graph. C) Comparison of the highest transfection efficiency in plasmid B to equal ratios achieved with plasmid A. The ratio of DNA/PEI [ng/ng] is indicated directly below the columns. Statistical significance is indicated (*p < 0.05, **p < 0.01). Comparisons were made in the grouped ratios. N = 6 with n = 3. Data in S4 Dataset.

In cells treated with a transfection reagent consisting of plasmid A and PEI, successfully transfected cells were observed in every tested ratio (Fig 5A). The highest percentage of transfected cells was 0.4 ± 0.58% with DNA 1000 ng and PEI 500 ng. The remaining ratios resulted in percentages between 0.35 ± 0.54% (250 ng/250 ng) and 0.01 ± 0.033% (1000 ng/250 ng).

Better results were achieved with transfection reagents consisting of plasmid B and PEI, again with every tested ratio showing a number of transfected cells (Fig 5B). However, the highest percentage of transfected cells was 9.18 ± 3% with DNA 1000 ng and PEI 1000 ng. For 250 ng of DNA, the highest percentage was 8.32 ± 4.19% with PEI 500 ng, and for 500 ng DNA, the highest transfection rate was 6.79 ± 4.69% with 500 ng PEI. For the remaining ratios, the efficiency ranged between 0.03 ± 0.07% (500 ng/2000 ng) and 4.46 ± 1.93% (500 ng/1000 ng).

When comparing plasmid A to plasmid B (Fig 5C), significant differences were only present in DNA/PEI 250 ng/500 ng (p = 0.008), 500 ng/500 ng (p = 0.02) and 1000 ng/1000 ng (p = 0.005).

## Discussion

The aim of this study was to establish a reliable, non-viral method for transfection of recombinant vectors into fibroblasts, which can potentially transferred to be incorporated in a coating of cochlear implants. Since fibroblast growth around the implant is an unavoidable consequence of the implantation process [15], the final goal is to integrate plasmid DNA carrying a gene coding for BDNF into endogenous fibroblasts. This should result in the secretion of the growth factor, thus enhancing the survival of the SGN and their neuritogenesis. Currently, only limited reports on transfection of fibroblasts exist [36,37]. Although ear-specific fibroblasts were available [39,40], NIH/3T3 fibroblasts were chosen since they represent a well characterized and reliable cell line and have been successfully transfected earlier to produce BDNF [35]. PEI is the most commonly investigated cationic polymer and ‘gold standard’ for cationic transfection [24]. PEI exists in linear and branched variants, with the main difference being the presence of primary and tertiary amino groups in branched PEI in addition to the secondary amino groups in linear PEI [33]. These result in a tighter complex formation and higher buffering capabilities [41] of branched PEI, but also smaller complexes [33].

The efficiency of transfection is dependent on the cellular uptake mechanism of the target cells. The main uptake mechanism for DNA-PEI polyplexes is endocytosis [33], the efficiency of which is partially determined by the size and surface charge of the polyplexes [42], amongst other factors. Typically, smaller sizes and a positive charge are beneficial for successful transfection [42]. Both properties can be determined via dynamic light scattering and electrophoresis, respectively. A change in particle size and zeta potential can therefore not only prove the formation of DNA-PEI-complexes, the requirement for successful transfection, but also provide tendencies towards the efficiency of the process, since a smaller particle size of around 50 nm is deemed optimal [42]. Nevertheless, results in particle size and surface charge determination should be carefully challenged since both, particle size and zeta potential, can be impaired by certain factors due to the nature of the measuring process, which can result in high standard deviation. Firstly, both measurements are dependent on the concentration of the sample in the tested solution [43]. The minimum concentration is dependent on the sample (e.g., 0.1 mg/ml for a 15 kDa protein) [44], and while our concentrations (250 ng/100 µl up to 2000 ng/100 µl) were sufficient to gain adequate results, a higher concentration and therefore more particles may result in lower standard deviation. Since the test solution was injected into the test chambers instead of filling the entire cuvette with transfection solution, diffusion could have reduced particle concentration at the spot of measurement. The presence of PBS does provide an unavoidable impairment as well. Both, the amount of salt ions in the transfection solution and the pH-value affect particle size and zeta potential [43,45]. However, solution in PBS is necessary as all substances were stored in stock solutions in PBS. In addition, accumulation of particles can result in multiple notable peaks [43]. Depending on the amount and ratio of DNA and PEI, this was observed in our results and does influence the mean particle size as well. Lastly, the sign of the zeta potential cannot reliably be measured using electrophoresis [43], but requires further titration.

Particle size and zeta potential of DNA/PEI-particles have been measured in earlier studies, with particle sizes commonly ranging from 80–100 nm [29,30,36,37] up to 200–400 nm [28–30,46]. In other studies, sizes up to 2000 nm were reported [36,45]. This is in accordance to our findings with particle sizes between 95–206 nm. A trend towards larger particle sizes in DNA-PEI-complexes in comparison to equal amounts of DNA or PEI alone indicates a successful formation of particles in both plasmid A and plasmid B. Furthermore, comparing the particle size of polyplexes of plasmid A and B in equal DNA/PEI ratios shows a trend towards smaller particle size in polyplexes of plasmid B, despite the fact that results were mostly not found statistically relevant. Furthermore, measured values for PBS appear to be not realistic. The high standard deviation in these measurements indicates difficulties of the system to determine the size correctly. The size of the different ions would be at or below the detection limit of the zetasizer of 0.3 nm. This interpretation is supported by the fact that with larger particle sizes as measured in the current study in the DNA-PEI complexes, the standard deviation becomes much smaller.

While no significant differences between the individual DNA/PEI ratios or the different plasmids were detected, results of the current study of the zeta potential were in concordance with earlier findings demonstrating zeta potential ranges between −41 mV [29] and 40 mV [46] indicating large diversity in absolute value and charge [30,37]. The measured charge after addition of PEI to form DNA-PEI-complexes was reversed to the charge measured for DNA alone, indicating a change of zeta potential as described elsewhere [46] concluding successful complex formation, which is mandatory for successful transfection.

The potential cytotoxicity of both PEI [30] and DNA-PEI-complexes has been described before [47] and has to be considered for transfection procedure. While reducing the fibrous tissue growth around cochlear implants has been proven to be beneficial to reduce electrode impedance [17], reduction of fibroblasts was not the goal of the transfection and could potentially reduce transfection efficiency or the amount of finally released BDNF. Therefore, cell survival had to be investigated. For this reason, MTT tests were used to determine metabolic activity in cells treated with DNA, PEI or DNA-PEI-complexes. In accordance with earlier findings, DNA alone did not show an effect on cellular activity [29,36]. PEI, however, showed a decrease in metabolic activity with increasing amount of PEI in the well. This is in accordance to earlier reports [30], when branched 25 kDa PEI was used on A431 cells, showing an LD50 of 37 µg per well on a 96-well plate. The difference in LD 50 compared to our findings is influenced by the different cell line used and different incubation period (4 h, compared to 3 h in our tests). An amount of 2000 ng PEI per well exhibited a metabolic activity of 14.08% compared to untreated cells. A significant decrease in cellular activity could also be measured in DNA-PEI-complexes of plasmid A with a DNA/PEI-ratio of 250 ng/1000 ng, which was not present in the same ratio in plasmid B. Metabolic activity in the remaining DNA-PEI-complexes was not reduced. Earlier reports have already shown decreased cytotoxicity of PEI when combined with plasmid DNA [30], making a successful transfection using DNA-PEI-complexes possible. The single difference may be a result of the size difference of the plasmids. Longer plasmid DNA has been shown to result in a higher cytotoxicity in target cells when using electrotransfer [48].

Expectedly, control experiments showed that transfection with either DNA or PEI alone did not yield any transfected cells. In contrast, every tested ratio of DNA/PEI with both plasmids showed tdTomato-positive cells indicating successful transfection. The transfection efficiency ranged between 0.1% and 9.18%, which is little lower than earlier findings of 20% transfection efficiency using 3T3-fibroblasts and PEI [37]. Transfection with an amount of 2000 ng PEI per well never yielded a higher rate of transfection than 1%, regardless of the amount or type of DNA used. Transfection with PEI results only in a transient transfection. Cells might lose tdTomato fluorescence after some time and other cells might only be detectable after more than 24 h. This might also explain weak and strong fluorescence in Fig 4A. Therefore, the detected transfection efficiency might be some kind of minimum transfection rate. Further experiments have to address this question more deeply.

With the above mentioned lower metabolic activity resulting from high amounts of PEI per well, this matches previous reports of reduced cellular survival in lower DNA/PEI ratio [28]. The ratio of DNA to PEI has been shown to influence the transfection efficiency in different cells lines (including NIH/3T3 fibroblasts) [36,37], which is present in our results in NIH/3T3-fibroblasts as well.

With plasmid A, the highest transfection efficiency achieved was 0.4, whereas with plasmid B it was 9.18%. As both plasmids used different promotors, this could potentially be a reason for the observed difference. Both promotors lead to high gene expression especially in eukaryotic cells. However, depending on the cell type, there are reports stating SFFV is much more efficient than CAG [49] or that CAG is more effective than SFFV [50]. As we are not aware of a comparison of both promotors in fibroblasts, the choice of promotors could have influenced transfection efficiency in our study. Another possible explanation might be the difference in lengths between the two plasmids, with plasmid B having roughly two thirds of the length of plasmid A. This hypothesis is further supported by a report of an even higher transfection efficiency of 25% [37] in NIH/3T3-fibroblasts using PEI, in which the used plasmid of 4731 bp length [51] was even shorter than plasmid B. These findings suggest a correlation between plasmid length and expected transfection efficiency, meaning a shorter plasmid would be expected to yield better results than a larger plasmid. When electroporation was used as means of transfection, several reports show such a correlation between plasmid length and transfection efficiency [48,52,53], going as far as to establish an optimal length for this specific method (between 1,869–4,257 bp) [52], although variations depending on cell type could be a factor. Similar effects were reported using cationic transfection with lipopolyamine in NIH/3T3 [54], with a smaller plasmid size resulting in better transfection efficiency. Further research, again using lipopolyamine and NIH/3T3 cells [47], has shown no size difference in complexes of plasmids with different lengths. However, there was still an improved transfection efficiency in smaller plasmids. The deciding factor in this method of transfection did not seem to be particle size, but intracellular mechanisms, which benefit from a smaller plasmid with fewer base pairs [47]. Our findings, while not showing significant differences in particle size between complexes with equal DNA/PEI-ratio and different plasmids, produced a better transfection efficiency with the smaller plasmid B. It can be speculated that plasmid size does have an influence on transfection efficiency also in polycationic transfection with PEI without showing significant differences in particle size. Further research will be needed to confirm a connection.

The presented study can only be a first step on the way to transfect fibroblasts on the surface of cochlear implants. The system has to work with ear-specific fibroblasts and has to be included in a coating on the CI electrode. May be for in vivo application one could skip the DNA sequence for the fluorescent marker protein and make the plasmid even smaller. For the current in vitro experiments, the marker protein just simplified the investigation. Finally, the success of the approach will depend on the amount of BDNF delivered from the transfected cells and the duration of BDNF production, both of which have to be addressed in future studies.

## Conclusion

The changes in particle size and zeta potential indicate a successful formation of complexes with both plasmids used. The findings testify to a large influence of plasmid length on transfection efficiency in cationic polymer transfection using PEI as transfection agent. With successful transformation of nearly 10% of cells and a comparatively low cytotoxicity, the proposed transfection system using plasmid B may be used in further experiments.

## Supporting information

S1 Dataset Partcle size. This is the dataset behind Fig 1.
(XLSX)

S2 Dataset Zeta potential. This is the dataset behind Fig 2.
(XLSX)

S3 Dataset MTT test. This is the dataset behind Fig 3.
(XLSX)

S4 Dataset Transfection efficiency. This is the dataset behind Fig 5.
(XLSX)
